# Possible Link Between Macrocytosis and Hypertriglyceridemia: A Case Report

**DOI:** 10.7759/cureus.92455

**Published:** 2025-09-16

**Authors:** Jeremy I Purow, Joseph Luzarraga, Aron Berkman

**Affiliations:** 1 Hematology and Oncology, Florida International University, Herbert Wertheim College of Medicine, Miami, USA

**Keywords:** anemia, hypertriglyceridemia, lipid uptake, macrocytosis, red blood cell

## Abstract

Macrocytosis is relatively common and can be due to various etiologies. More common causes include vitamin B12/folate deficiency, liver disease, alcohol use, hypothyroidism, and myelodysplastic syndrome. A work-up often yields the etiology, but in some patients, it remains elusive. We present a case of a 75-year-old female with persistent and unexplained macrocytosis. A work-up for the common causes was unremarkable. However, she had a markedly elevated triglyceride level. This report implies a possible association between hypertriglyceridemia and macrocytosis. Further study is warranted to explore this relationship and red blood cell uptake of triglycerides.

## Introduction

The workup of anemia follows well-described algorithms. Anemia may be described as hyperproliferative, exemplified by reticulocytosis, where there is a reaction of the bone marrow to excessive loss or destruction of red cells [1]. All other causes of anemia are described as hypoproliferative, characterized by failure of the bone marrow to efficiently produce new red blood cells [1]. The etiologies of hypoproliferative anemia are broad [1]. The workup of hypoproliferative anemias is based on a morphologic algorithm centered around red cell size, as well as a review of red cell morphology on the peripheral blood smear [1]. Red cell size is characterized by three categories: microcytic (mean corpuscular volume, or MCV < 80 fL), normocytic (MCV 80 to 96 fL), and macrocytic (MCV > 96 fL) [1]. 

There are two categories of macrocytosis. Megaloblastic anemia is a subset within this category and is associated with faulty DNA synthesis, resulting most commonly from folic acid or vitamin B12 deficiency, and less commonly from the use of medications that affect DNA synthesis, such as chemotherapy and HIV drugs [1]. Morphologically, megaloblastic macrocytosis is manifested in the peripheral blood by macroovalocytosis and hypersegmented neutrophils, and in the bone marrow as abnormal maturation of red cell and neutrophil precursors (nuclear/cytoplasmic dyssynchrony), and is associated with giant band forms [1]. Non-megaloblastic macrocytosis is caused by disorders thought to be associated with abnormalities (usually acquired) in the red cell membrane, such as chronic liver disease, hypothyroidism, alcohol, and the hypoplastic marrow syndromes, such as myelodysplasia [1]. Morphologic changes in the peripheral blood include the appearance of round macrocytes, spiculated red cells (if chronic liver disease is present), or signs of dysplasia, particularly in the neutrophil (hyposegmented nuclei or hypogranulated cytoplasm) [1]. The bone marrow will not appear megaloblastic [1]. 

Other causes of macrocytosis include reticulocytosis (the reticulocyte is a young, larger red cell precursor). In addition, cold agglutinins, leukocytosis, and hyperglycemia have been associated with macrocytosis [1]. 

The workup of the patient with macrocytosis, therefore, begins with a reticulocyte count and evaluation of the peripheral blood smear, followed by a careful history (including medications) and physical examination [1]. Other laboratory work would include folic acid and vitamin B12 levels, liver function studies, thyroid function studies, and, when appropriate - such as in cases of multiple cytopenias or the suggestion of dysplastic changes in the peripheral blood - a bone marrow examination [1]. 

Despite a meticulous workup for macrocytosis, following the above algorithms, the etiology of macrocytosis will not be identified in as many as 10% of patients [2]. One study described 43 patients with unexplained macrocytosis [2]. Thirty-one patients had stable macrocytosis or resolution of the elevated MCV [2]. Twelve patients, however, developed a primary bone marrow disorder or a cytopenia, suggesting the need for careful follow-up of any patient with macrocytosis of unknown etiology [2]. Importantly, this study suggests that some patients with macrocytosis have an unknown etiology [2].

Our report describes a patient with a thorough workup for macrocytosis that was unremarkable. Her only significant abnormality was elevated triglycerides. This led us to posit a possible relationship between her macrocytosis and triglyceride levels, due to increased lipid uptake in the red blood cell membrane.

## Case presentation

This is a 75-year-old female who was referred by her primary care provider for evaluation of macrocytosis. She had a history of hyperlipidemia, osteoporosis, vitamin D deficiency, pulmonary nodules, and gastroesophageal reflux disease. Her medications were alendronate 70 mg once weekly, atorvastatin 20 mg once daily, buspirone 10 mg twice daily, and gabapentin 300 mg three times daily. She had no specific family history of hematological issues. The patient’s physical examination was unremarkable.

Her review of systems was unremarkable. Notably, she had no fatigue, weakness, or other symptoms of anemia or other blood abnormalities. The patient reported that she drinks one or two glasses of wine, whiskey, or beer per week. On general inspection of the patient, she was a healthy-appearing, well-nourished, alert, elderly woman in no acute distress. Her physical examination was unremarkable. A complete blood count (CBC) at presentation was obtained (Table 1).

**Table 1:** Initial Workup for Macrocytosis Complete blood count (CBC), folate, vitamin B12, thyroid-stimulating hormone (TSH), free T4, liver function tests, hepatitis panel, *Helicobacter pylori* breath test, and reticulocyte count were within normal limits.

Lab Test	Result	Reference Range
White blood cell count	5.7 × 10³/µL	3.8-10.8 × 10³/µL
Red blood cell count	3.79 × 10⁶/µL	3.80-5.10 × 10⁶/µL
Hemoglobin	12.9 g/dL	11.7-15.5 g/dL
Hematocrit	37.5%	35.0-45.0%
Mean corpuscular volume (MCV)	98.9 fL	80.0-100.0 fL
Mean corpuscular hemoglobin (MCH)	34.0 pg	27.0-33.0 pg
Mean corpuscular hemoglobin concentration (MCHC)	34.4 g/dL	32.0-36.0 g/dL
Red cell distribution width (RDW)	11.8%	11.0-15.0%
Platelet count	206 × 10³/µL	140-400 × 10³/µL
Mean platelet volume (MPV)	10.8 fL	7.5-12.5 fL
Differential	Normal	-
Folate	12.1 ng/mL	>5.4 ng/mL
Vitamin B12	850 ng/mL	200-1,100 pg/mL
Thyroid-stimulating hormone (TSH)	2.82 mIU/L	0.4-4.5 mIU/L
Free T4	0.9 ng/dL	0.8-1.8 ng/dL
Aspartate aminotransferase (AST)	19 U/L	10-35 U/L
Alanine aminotransferase (ALT)	20 U/L	6-29 U/L
Hepatitis (A, B, C)	Negative	Negative
*Helicobacter pylori* breath test	Negative	Negative
Reticulocyte count	79,590 cells/µL	20,000-80,000 cells/µL

The initial CBC showed an MCV at the upper limit of normal. The only other significant abnormality during the patient's workup was abnormal lipid panels. Specifically, triglycerides and non-high-density lipoprotein (non-HDL) cholesterol were elevated at multiple timepoints. MCV values at other timepoints were also elevated. Table 2 presents the MCV and triglyceride levels at specific timepoints.

**Table 2:** Mean Corpuscular Volume and Lipid Data All the mean corpuscular volumes were elevated or close to the upper limit of normal. Triglyceride levels were significantly elevated. Non-HDL cholesterol was significantly elevated on multiple occasions, but not consistently. HDL, high-density lipoprotein

Date	Triglycerides (mg/dL) (Reference Range: <200 mg/dL)	Mean Corpuscular Volume (fL) (Reference Range: 80-96 fL)	Non-HDL Cholesterol (mg/dL) (Reference Range: <190 mg/dL)
7/23/2020	448 mg/dL	100.3 fL	230 mg/dL
9/17/2020	197 mg/dL	-	108 mg/dL
3/18/2021	256 mg/dL	-	122 mg/dL
8/31/2021	258 mg/dL	-	148 mg/dL
2/10/2022	226 mg/dL	99.5 fL	121 mg/dL
8/31/2022	-	102.3 fL	153 mg/dL
1/19/2023	-	99.5 fL	122 mg/dL
5/11/2023	-	100.8 fL	125 mg/dL
8/9/2023	379 mg/dL	107.9 fL	133 mg/dL
9/13/2023	-	98.9 fL	-
12/16/2023	310 mg/dL	99.2 fL	127 mg/dL

## Discussion

The red blood cell is a biconcave disk that has an area of central pallor and a fluid-like double membrane. The membrane contains a complex and asymmetric arrangement of lipids, carbohydrates, and proteins [3-5]. The lipids within the red blood cell membrane are both phospholipids and cholesterol, which are distributed throughout the bilayer [6]. The membrane contains uncharged and charged variants of phospholipids, including phosphatidylcholine, sphingomyelin, phosphatidylethanolamine, and phosphatidylserine [6]. Due to the absence of membrane-bound organelles in red blood cells, they are unable to make their own lipids [6]. Additionally, it was conventionally thought that triglycerides would not be stored in red blood cells, since red blood cells lack mitochondria to metabolize them. However, a recent study by researchers at the Mayo Clinic found that (U-13C)palmitate, a radiolabeled free fatty acid, was incorporated into red blood cells in vitro as triglycerides [7]. This suggests that red blood cells may incorporate fatty acids, which are structurally similar to phospholipids, into their cell membranes [7].

We hypothesize that the macrocytosis seen in our case was caused by markedly elevated hypertriglyceridemia, as the excess triglycerides, in the form of free fatty acids, were incorporated into the cell membranes of red blood cells, making them larger [7]. An extensive workup for the typical etiologies of macrocytosis - including folate and B12 deficiencies, bone marrow disorders (suggested unlikely by an otherwise normal CBC), alcoholism, and liver disease - was unrevealing.

We did consider the fact that the patient’s non-HDL cholesterol levels were also slightly elevated at certain points, and these levels may have contributed to her macrocytosis as well. However, we do not believe that these elevated cholesterol levels played a major role, due to the fact that her cholesterol levels were only slightly elevated at most time points, and the fluctuation of her cholesterol levels does not correlate with her MCV [7]. Despite mild alcohol consumption, she had normal liver function tests, making this etiology for the macrocytosis less likely.

We believe that the hypothesis regarding the correlation between triglyceride levels and MCV may have multiple diagnostic and clinical implications. It is conceivable that, of the 10% of patients with macrocytosis of unclear etiology, as cited above, a new subcategory - macrocytosis of hypertriglyceridemia - could be created. It is important to note that our analysis is based on a case report. Further retrospective studies, including patients with macrocytosis of unknown etiology, will be required, however, and are currently underway. It is important to conduct a more extensive study in patients with elevated triglyceride levels to determine whether a correlation between triglycerides and MCV exists in the broader patient population. If a relationship is ascertained, MCV may need to be adjusted based on triglyceride levels in the blood.

## Conclusions

Our case report demonstrates a possible relationship between macrocytosis and hypertriglyceridemia. If further studies confirm the relationship, additional preclinical studies would be warranted to further explain this relationship, as well as a possible relationship between hypertriglyceridemia and the cell membranes of other cell types. For now, however, any patient who presents with unexplained macrocytosis requires cautious long-term follow-up in order to rule out the possibility that a significant clinical diagnosis is evolving.

## References

[1] Kaferle J, Strzoda CE (2009). Evaluation of macrocytosis. Am Fam Physician.

[2] Younes M, Dagher GA, Dulanto JV, Njeim M, Kuriakose P (2013). Unexplained macrocytosis. South Med J.

[3] Li H, Lykotrafitis G (2014). Erythrocyte membrane model with explicit description of the lipid bilayer and the spectrin network. Biophys J.

[4] Lux SE (2016). Anatomy of the red cell membrane skeleton: unanswered questions. Blood.

[5] Steck TL (1974). The organization of proteins in the human red blood cell membrane. A review. J Cell Biol.

[6] Ways P, Hanahan DJ (1964). Characterization and quantification of red cell lipids in normal man. J Lipid Res.

[7] Song Y, Jensen MD (2021). Red blood cell triglycerides-a unique pool that incorporates plasma-free fatty acids and relates to metabolic health. J Lipid Res.

